# Mechanistic insight into spontaneous transition from cellular alternans to arrhythmia—A simulation study

**DOI:** 10.1371/journal.pcbi.1006594

**Published:** 2018-11-30

**Authors:** Wei Wang, Shanzhuo Zhang, Haibo Ni, Clifford J. Garratt, Mark R. Boyett, Jules C. Hancox, Henggui Zhang

**Affiliations:** 1 Biological Physics Group, School of Physics & Astronomy, The University of Manchester, Manchester, United Kingdom; 2 School of Computer Science and Technology, Harbin Institute of Technology, Harbin, China; 3 Manchester Heart Centre, Manchester Royal Infirmary, Manchester, United Kingdom; 4 School of Physiology, Pharmacology and Neuroscience, and Cardiovascular Research Laboratories, School of Medical Sciences, University of Bristol, Bristol, United Kingdom; 5 Key Laboratory of Medical Electrophysiology of Ministry of Education and Medical Electrophysiological Key Laboratory of Sichuan Province, Institute of Cardiovascular Research, Southwest Medical University, Luzhou, Sichuan, China; 6 Space Institute of Southern China, Shenzhen, China; Universiteit Gent, BELGIUM

## Abstract

Cardiac electrical alternans (CEA), manifested as T-wave alternans in ECG, is a clinical biomarker for predicting cardiac arrhythmias and sudden death. However, the mechanism underlying the spontaneous transition from CEA to arrhythmias remains incompletely elucidated. In this study, multiscale rabbit ventricular models were used to study the transition and a potential role of *I*_*Na*_ in perpetuating such a transition. It was shown CEA evolved into either concordant or discordant action potential (AP) conduction alternans in a homogeneous one-dimensional tissue model, depending on tissue AP duration and conduction velocity (CV) restitution properties. Discordant alternans was able to cause conduction failure in the model, which was promoted by impaired sodium channel with either a reduced or increased channel current. In a two-dimensional homogeneous tissue model, a combined effect of rate- and curvature-dependent CV broke-up alternating wavefronts at localised points, facilitating a spontaneous transition from CEA to re-entry. Tissue inhomogeneity or anisotropy further promoted break-up of re-entry, leading to multiple wavelets. Similar observations have also been seen in human atrial cellular and tissue models. In conclusion, our results identify a mechanism by which CEA spontaneously evolves into re-entry without a requirement for premature ventricular complexes or pre-existing tissue heterogeneities, and demonstrated the important pro-arrhythmic role of impaired sodium channel activity. These findings are model-independent and have potential human relevance.

## Author summary

T-wave alternans (TWA), manifested as beat to beat alterations between large and small T-wave amplitudes on the electrocardiogram (ECG) is one of the prevalent clinical observations that are closely associated with cardiac arrhythmias and sudden death. TWA is believed to be underlined by cardiac alternans at the cellular level, but the extract mechanism for the transition from cellular alternans to that at the tissue level, and how this further spontaneously evolves into cardiac arrhythmias remains incompletely elucidated. In this study, multiscale rabbit ventricular computational models were used to address this issue by investigating the underlying mechanism(s) for the arrhythmogenesis of cardiac alternans, as well as a possible role of sodium channel on perpetuating cardiac arrhythmias. Our results demonstrated a spontaneous development of re-entry from cellular alternans, arising from a combined action of APD and CV restitution properties with the curvature-dependence of CV. Tissue inhomogeneity and anisotropy further promoted break-up of excitation waves, leading to multiple re-entrant excitation waves. It was also found impaired sodium channel with either increased or decreased channel current facilitated the arrhythmogenesis. This study provides new insights into underlying the mechanism, by which cellular cardiac alternans spontaneously evolves into cardiac arrhythmias. Similar results were observed in human atrial tissue models, suggesting our major findings are model-independent and of potential clinical relevance.

## Introduction

Cardiac alternans is comprised of beat-to-beat alterations in cardiac electrical and mechanical activities [1]. At the cellular level, cardiac electrical alternans (CEAs) manifests as alterations either in the duration of the action potential (APD alternans) or/and in the cytosolic calcium transient amplitude (CaT alternans) [2]. Clinically, cardiac alternans especially that associated with the APD alternans can be detected as electrocardiographic T-wave alternans (TWA), which has been recognised as a biomarker for predicting the onset of cardiac arrhythmias and sudden cardiac death (SCD) [3–5]. As TWA is associated with increased risk of cardiac arrhythmogenesis in many heart diseases, such as heart failure [6], ischemia [7] and long QT syndromes [8,9], it is crucially important to understand possible underlying mechanism(s) of arrhythmogenesis in association with cardiac alternans.

Previous experimental and simulation studies have unravelled possible mechanisms underlying the onset of cardiac alternans [10,11]. One of the most well-known hypotheses for the genesis of APD alternans is the APD restitution theory, first established by [12], which theoretically attributes the generation and sustainability of cardiac alternans to the slope of APD restitution curve. When the maximal slope of the APD restitution curve is greater than 1, then sustained APD alternans can be produced at fast pacing rates. This theory has been supported by some experimental and simulation studies (e.g. [13–15]). However, due to the effect of cardiac excitation memory, some other studies [16–18] have found that the APD restitution theory is not sufficient to produce stable alternans and more complicated dynamic processes are involved. Another theory is about the primary role of CaT alternans, which may be generated by a stiff relationship between the Ca^2+^ content in the SR and calcium release from the Ryanodine receptors (RyRs) [18,19]. By mechano-electrical coupling [20], such CaT alternans may be reflected as APD alternans, then reflected as TWA. This theory is supported by some experimental studies in observing CaT alternans with flat APD restitution curve or under voltage clamp conditions [19,21,22]. APD alternans promoted by CaT alternans may account for cardiac alternans at slow pacing rates [23].

At the tissue level, cellular APD alternans can be manifested as spatially concordant and/or discordant excitation wave alternans [24]. With concordant alternans, the whole tissue exhibits uniform alternation of long or short APD of one excitation waves. However, with discordant alternans, non-uniform distribution of long and short APD within the tissue at the same excitation wave can be seen. Several mechanisms have been proposed to be responsible for the formation of discordant alternans [25,26]. One is the intrinsic heterogeneous electrical properties of the tissue, by which cells in different regions have different repolarisation properties (such as epicardium to endocardium heterogeneity, or apex to base heterogeneity) [24]. Such heterogeneous electrical properties of tissue may produce out-of-phase excitation waves manifested as discordant alternans. In conditions with a decreased inter-cellular electrical coupling (e.g. due to fibrosis), individual cells’ intrinsic heterogeneous properties can be preserved due to reduced electronic interactions from neighbouring cells, enabling tissue to generate discordant alternans [27,28]. However, other studies found intrinsic heterogeneity of tissue is an unnecessary condition for the genesis of discordance [29–31]. Watanabe *et al*. [29] demonstrated that discordant alternans can be developed without spatial inhomogeneity in electrophysiology due to the nature of conduction velocity (CV) restitution, which has also been observed in other studies [30,31]. In the case when discordant alternans is associated with unstable dynamics of intracellular calcium cycling, no contribution of CV restitution is required [32,33].

Discordant alternans is believed to be strongly arrhythmogenic as it may increase spatial dispersion of refractoriness, thus facilitating uni-directional conduction block and leading to formation of re-entry [24,30]. However, due to the complexity of cardiac excitation and propagation, the mechanism by which APD alternans at the cellular level spontaneously evolves into re-entrant excitation waves at the tissue level remains incompletely elucidated.

TWA has been known to be associated with the repolarisation phase of the action potential, during which L-type calcium and various potassium channel currents play important roles [5,34–36]. However, it is also associated with the depolarisation phase [37–39], during which the sodium channel current (*I*_*Na*_) plays an important role in determining the maximal upstroke velocity of action potentials. It has been found that malfunction of *I*_*Na*_ is associated with various cardiac conduction diseases [40,41], modulates the effective refractory period between cardiac action potentials [42], which may also promote the genesis of alternans at the cellular level and discordant alternans at the tissue level. So far, the role of *I*_*Na*_ in perpetuating the genesis of discordant alternans leading to formation of re-entry has not been completely elucidated.

In this study, we used a simulation approach to investigate the mechanism(s) by which cardiac alternans in a single cell transitions spontaneously into re-entrant arrhythmia in both homogeneous and heterogeneous cardiac tissues. The role of *I*_*Na*_ in generating and transitioning the APD alternans to discordant alternans that perpetuates re-entrant excitation waves was also investigated.

## Methods

### Single cell model and AP simulations

The rabbit ventricular epicardial cell model developed by Aslanidi et al. [43] was used in this study. This model was chosen as it is able to generate stable large and small AP alternations at pacing cycle length < 188 ms, and is suitable for long time period simulations. At the cellular level the typical Hodgkin-Huxley model of a cardiac cell was implemented, by which the cell membrane is modelled as a capacitor aligned in parallel with ion channel currents, Na^+^-Ca^2+^ exchangers and Na^+^-K^+^ pumps that are responsible for generating cardiac action potentials. In brief, the model equation is represented as:

$$\frac{\partial V}{\partial t}=-\frac{I_{ion}+I_{stim}}{C_{m}}$$ (1)

where *V* is the membrane potential, *t* the time, *I*_ion_ the sum of all transmembrane ionic currents [43], *I*_stim_ the externally applied stimulus current and C_m_ the cell capacitance per unit surface area.

In order to investigate the role of *I*_*Na*_ in the generation of alternans and its transition into re-entrant excitation waves, a scaling factor (*S*_*gNa*_) was used to modulate the macroscopic conductance of the sodium channel by the following equation:

$$I_{Na}=S_{g_{Na}}g_{Na}m^{3}hj\left(V_{m}-E_{Na}\right)$$ (2)

where *g*_*Na*_ is the maximal channel conductance, *m* the activation gate, *h* and *j* the fast and slow inactivation gate respectively [44], *V*_m_ the membrane potential, and *E*_Na_ the channel’s reversal potential [43].

In order to allow the cell model to generate AP alternans at a wide pacing cycle length (PCL) range, the time constant of the inactivation gate *h* of the *I*_*Na*_ channel was increased by 30ms at membrane potentials negative to -70mV, mimicking a prolonged recovery time for the *I*_*Na*_ channel as seen in some familial arrhythmia syndromes associated with sodium channel dysfunction caused by mutations in the SCN5A gene (for details see a review in [45]) (see Online Supplement Material S1 Text for details). In order to characterise fully the effects of varying the recovery time of *I*_*Na*_ on alternans genesis and conduction, simulations with graded increases in the time constant of the inactivation of *h*, ranging from 0 to 40 ms, were also conducted.

### Tissue model for AP propagations

Multicellular tissue model for simulating the AP propagation was based on the well-established mono-domain equation [46]:

$$\frac{\partial V}{\partial t}=\nabla \cdot (D\nabla V)-\frac{I_{ion}}{C_{m}}$$ (3)

where **D** is the diffusion coefficient matrix determining the AP's conduction velocity in tissue.

For one-dimensional (1D) simulations, it can be presented as:

$$\frac{\partial V}{\partial t}=D\left(\frac{\partial^{2}V}{\partial x^{2}}\right)-\frac{I_{ion}}{C_{m}}$$ (4)

where the diffusion coefficient *D* is a scalar value.

For 1D simulations, a ventricular strand of a total length of 120 mm was discretised by a spatial resolution of 0.15 mm to form 800 interconnected nodes, each of which was modelled by the Aslanidi *et al*. cell model. In the model, the diffusion coefficient *D* was set to 0.15 mm^2^/ms, giving a conduction velocity (CV) of planar excitation waves of 57cm/s through the strand, which matches experimental data from the rabbit ventricles [47].

For two-dimensional (2D) simulations, an idealised geometry of cardiac tissue sheet with dimensions of 120×120 mm^2^ were used, which was discretised by a spatial resolution of 0.15 mm to form an 800×800 nodes discrete lattice. In isotropic tissue models, the diffusion coefficient **D** was set to be the same value as that used in the 1D simulation. In anisotropic tissue models, **D** for the direction in parallel to the fibre direction remains the same as that in the 1D, and for the direction perpendicular to the fibre was set be a quarter of that along the fibre, which gave a 2:1 ratio of the CV for along and perpendicular to the fibre according to the experimental data [48]. In order to consider the non-uniform anisotropic property of cardiac tissue, an idealised elliptical fibre orientation was implemented following a similar approach as used in [49] in the anisotropic 2D tissue by the following equation:

$$\theta \left(x_{0},y_{0}\right)=\arctan\left(-\frac{x_{0}}{4y_{0}}\right)$$ (5)

where (*x*_0_,*y*_0_) represents the coordinates of a point in the 2D tissue sheet with the origin being at the left-top corner, and *θ* denotes the fibre direction of the point.

### Simulation methods and protocols

At the single cell level, a steady-state protocol was used to evoke action potentials, from which cardiac alternans were analysed and the APD rate dependent curves were determined. In this protocol, a sequence of 20 supra-threshold stimuli with a fixed PCL was applied to the cell model to evoke APs until a steady-state was reached. Then the last two APs were recorded for analysis, for each of which the time interval between the upstroke of the AP and 90% repolarisation was measured as APD. The measured APD was plotted against variant PCLs to get the APD rate dependent curves. In addition, an S1-S2 protocol [50] was utilised to obtain the APD restitution curve. With the S1-S2 protocol, 20 S1 stimuli with a PCL of 800 ms and one extra S2 stimulus with decreasing diastolic intervals (DIs) were applied to the model. Then the APDs evoked by the S2 at various DIs were obtained for the APD restitution curves. Furthermore, the S1-S2 protocol was also used for computing the effective refractory period (ERP) rate dependent curves. For each simulation, the model was given a sequence of 20 S1 stimuli with a fixed PCL and one extra S2 stimuli with decreasing time intervals between the last two stimuli. Then the smallest time interval that could evoke an AP whose overshoot is over 0 mV was recorded and used to calculate ERPs. By varying the conditioning PCLs, the ERP rate dependence relationship was obtained by plotting the computed ERPs against PCLs.

Similarly, the same two protocols described above were used in 1D tissue models to obtain the CV rate dependence and CV restitution relationships respectively. In the 1D tissue model, excitation waves were evoked by a sequence of supra-threshold stimuli applied at one end of the strand. Then the CV was measured as the ratio between the distance and the excitation time interval between the 25^th^ and 75^th^ node to avoid effects from the boundary [51] and heterogeneous wave propagations at fast pacing condition. In the 2D tissue model, excitation waves were evoked by applying supra-threshold stimuli at the left-bottom corner of the tissue.

### Model-dependence test

To test possible model-dependence of simulation results and their human relevance, a well-established model for the human atrial action potentials developed by Courtemanche *et al*. (denoted as CRN model, [52]) was also implemented in cellular, 1D and 2D tissue models. Details of the model implementation and simulation protocols are documented in the Online Supplement S1 Text.

### Numerical details

At single cell level, Eq (1) and all gating variables were solved by Forward Euler (FE) method with a time step of *t* = 0.005 ms. At tissue level, Crank-Nicolson (CN) scheme [53] was implemented to solve Eq (3) for 1D and 2D simulations with a space step of *x* = 0.15 mm and a time step of *t* = 0.005 ms. At tissue boundaries, Neumann boundary conditions with zero-flux was implemented. All simulations were carried out on a system with 2 Intel Xeon E5 2680v2 10 core processors (40 logical cores) and 128 GB RAM memory, and OpenMP [54] was implemented for parallelising.

## Results

### Cellular AP alternans and role of *I*_*Na*_

**Fig 1:**
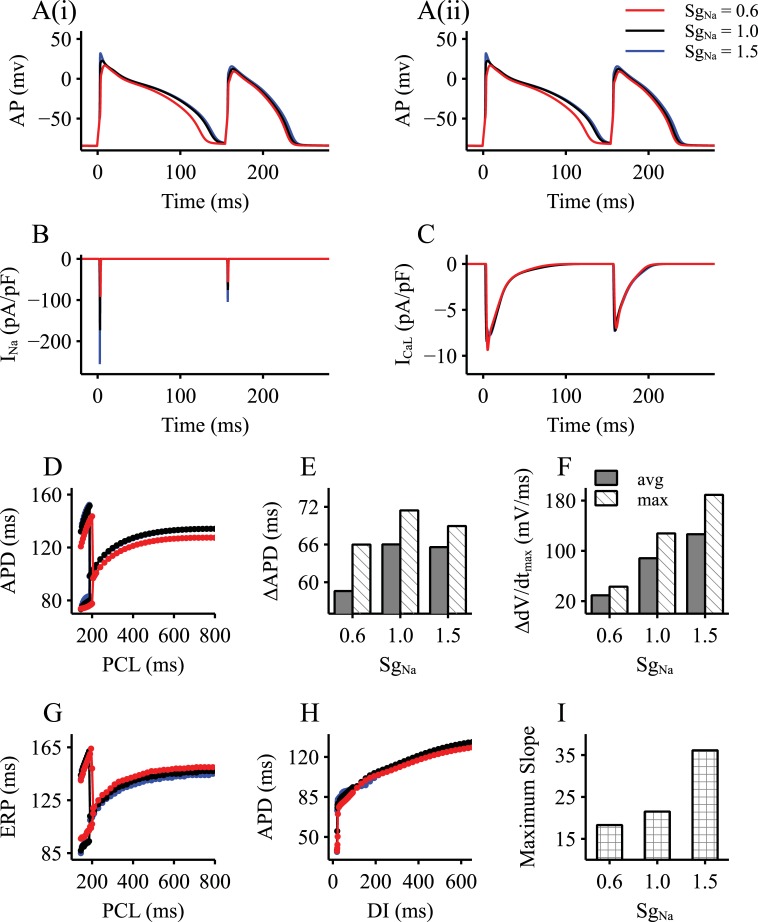
Cellular model properties with an altered *I*_*Na*_. Results were obtained by implementing three *S*_*gNa*_ values (*S*_*gNa*_ = 0.6, 1.0, 1.5), mimicking a reduced *I*_*Na*_, control and an increased *I*_*Na*_ respectively. A(i-ii). Representative AP alternans generated by modified rabbit EPI cell model. B. Recorded *I*_*Na*_ current traces during AP alternans. C. Recorded *I*_CaL_ current traces during AP alternans. D. Steady-state APD variations dependent on PCLs. E-F. The maximum and average differences of APD (E) and *dV/dt*_max_ (F) between the large and small AP during the range of PCLs that the model alters. G. ERP variations dependent on PCLs. H. APD restitution curves using S1-S2 protocol of the single cell models. I. The maximum slopes of the APD restitution curves in (H). DI: diastolic interval; PCL: pacing cycle length.

In response to a series of rapid stimuli using the steady-state protocol, the Aslanidi *et al*. model was able to generate AP alternans. Fig 1 shows a representative period of the time course of the simulated AP alternans with PCL at 160 ms (Fig 1A(i-ii)), during which distinctive APs showed alternating large and small amplitudes and durations. The underlying *I*_*Na*_ and *I*_CaL_ also showed significant large and small variations (Fig 1B and 1C), which were in phase with the AP variations during AP alternans.

Generation of the AP alternans was rate-dependent as shown in the computed APD restitution curve (Fig 1D). By decreasing PCL from 800 ms to 140 ms, a bifurcation point at PCL of 188 ms was observed, marking the PCL threshold for the genesis of AP alternans. Similarly, the computed ERP restitution curve also showed the bifurcation point at PCL of 188 ms, by which the ERP alternans was generated (Fig 1G).

Using the CRN model of the human atrial cell, obvious alternans in the action potential was also observed, which was associated with alternating *I*_*Na*_ and *I*_*CaL*_ as shown in the Online Supplement (S3.1 Fig).

The role of *I*_*Na*_ in modulating the profiles of large and small APs in their amplitudes and durations, as well as the PCL threshold for generating AP alternans was investigated. In Fig 1, simulation results with a reduced *I*_*Na*_ (*S*_*gNa*_ = 0.6) and increased *I*_*Na*_ (*S*_*gNa*_ = 1.5) on alternans were shown and compared with those in the control condition (*S*_*gNa*_ = 1.0). It was shown that reducing *I*_*Na*_ by 40% produced a marked effect on APD shortening at large PCLs; however, it only had a noticeable effect for the large AP of the alternans at small PCLs (Fig 1A–1C). It shifted the bifurcation point to the right, indicating a provocative role of *I*_*Na*_ reduction on the genesis of AP alternans, by which AP alternans was able to be generated by large PCLs (*i*.*e*., slow heart rates). *I*_*Na*_ reduction also reduced the APD difference (ΔAPD, Fig 1E) between the alternating large and small APs, so was the difference between the maximal upstroke velocities (Δ*dV/dt*_max_, Fig 1F). For the whole range of the PCL in the bifurcation area, the averaged differences of the APD and the maximal upstroke velocity between the alternating APs were also reduced by *I*_*Na*_ reduction. However, *I*_*Na*_ reduction increased the ERP at both large and small PCLs (Fig 1G), and reduced the maximal slope of the APD restitution curve obtained by the S1-S2 protocol (Fig 1H and 1I). It is interesting to note that *I*_*Na*_ reduction resulted in a shortened APD, but an increased ERP, implying a predisposing role of *I*_*Na*_ on arrhythmogenesis, as determined below.

On the other hand, an increased *I*_*Na*_ by 50% did not produce a noticeable effect on modulating the AP profiles for either the large or the small AP, although it decreased slightly the averaged difference of APD (ΔAPD), and increased that of the maximal upstroke velocity (Δ*dV/dt*_max_) between them (both measured locally at the bifurcation point and averagely over the whole bifurcation area) (Fig 1E and 1F). However, it increased the maximal slope of APD restitution curves computed from the S1-S2 protocol (Fig 1H and 1I). These results illustrated a higher degree of the difference between the large and small APs with an increased *I*_*Na*_ as compared to the control condition, which might also be associated with abnormal AP propagation in the tissue level.

**Fig 2:**
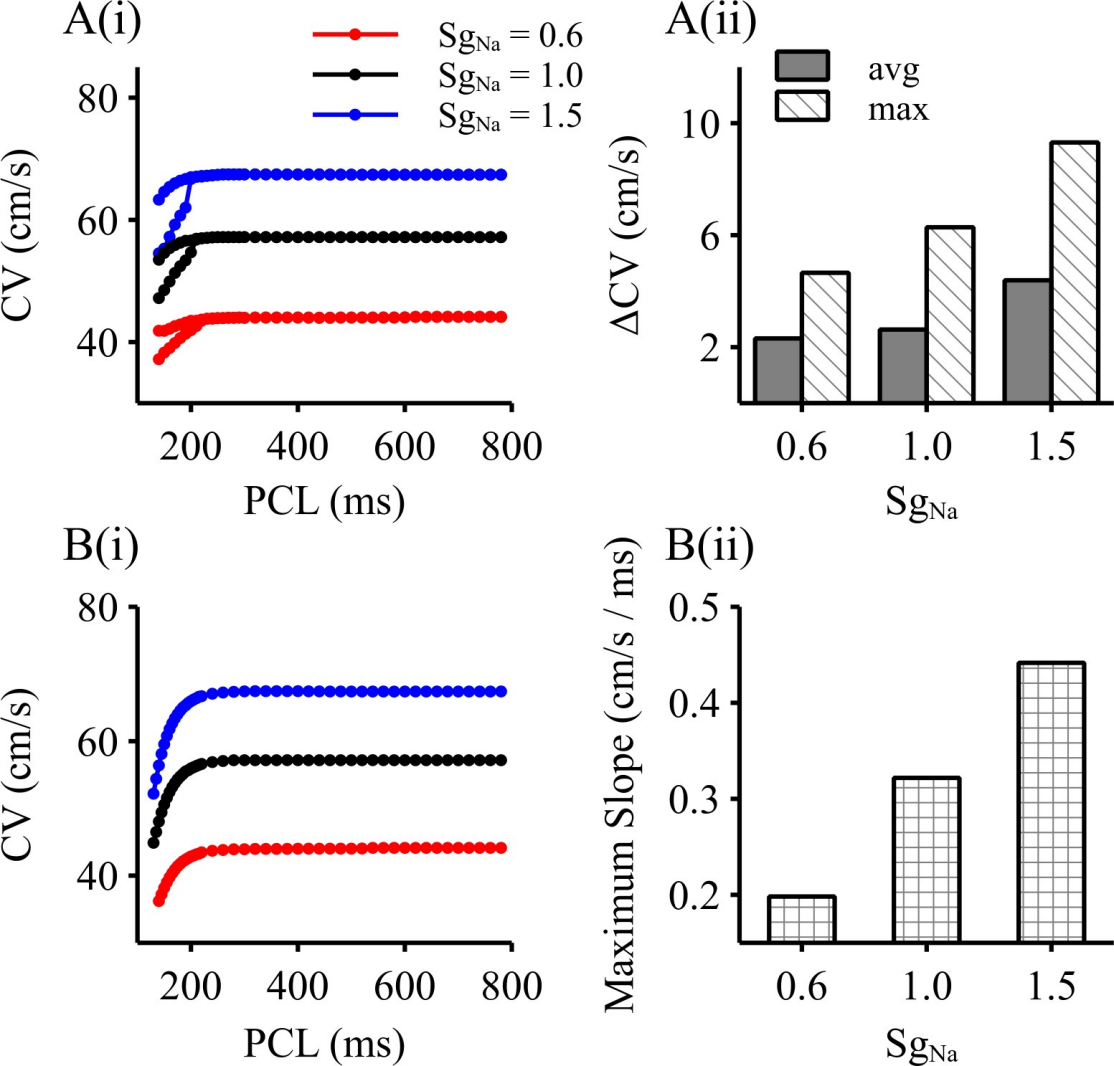
1D simulation results with an altered *I*_*Na*_. Results were obtained by implementing three *S*_*gNa*_ values (*S*_*gNa*_ = 0.6, 1.0, 1.5), mimicking a reduced I_Na_, control and an increased *I*_*Na*_ respectively. A(i). Computed PCL-dependent steady-state CV. A(ii). Maximal and averaged differences of CV between the fast and slow AP propagation during the range of PCLs producing conduction alternans. B(i). CV restitution curve computed by using the S1-S2 protocol. B(ii). The maximum slope of the CV restitution curve as shown in Fig B(i).

Alternating APs at the cellular level were reflected as alternans of AP conduction velocity as observed at the 1D tissue level. Fig 2 shows the computed conduction velocity (CV) restitution curves by the steady-state (Fig 2A) and the S1-S2 protocol (Fig 2B). As shown in Fig 2A(i), large and small CV alternations was generated at the PCL bifurcation point which was correlated with the one generating AP alternans, demonstrating that large and small APs were associated with fast and slow wave propagation in tissue respectively. It was also shown that a reduced *I*_*Na*_ resulted in a decreased difference of the CV between fast and slow conduction (Fig 2A(ii)), accompanying decreased maximal slope of the CV restitution (Fig 2B(ii)). On the other hand, an increased *I*_*Na*_ produced an increased difference of CV between fast and slow conduction, as well as an increased maximal slope of the CV restitution curve.

### Discordant alternans in 1D simulations

**Fig 3:**
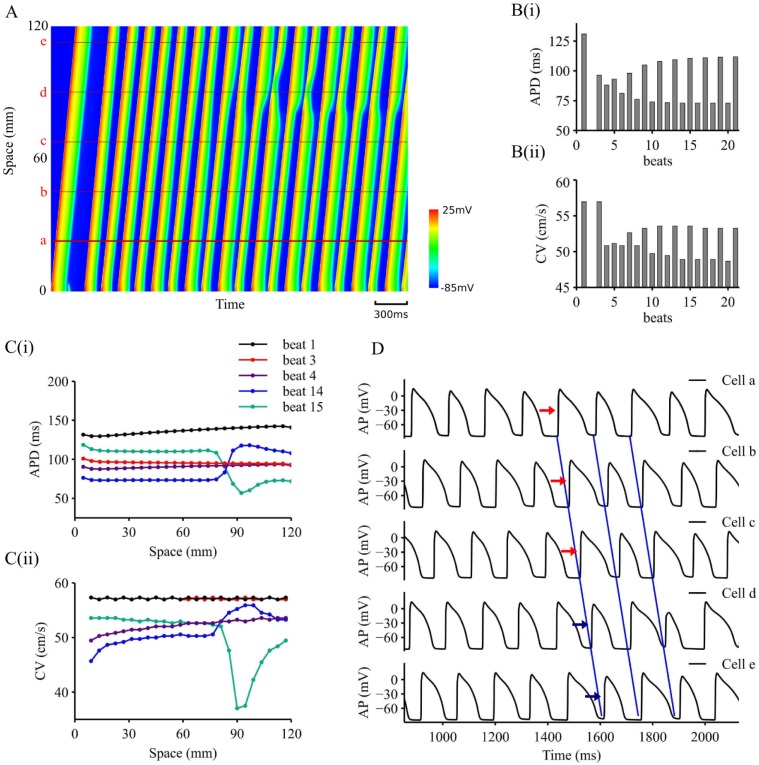
Representative 1D simulation results with *S*_*gNa*_ = 1.0, PCL = 140ms. A. Space-time plot of AP propagation on 1D strand. B. APD (B(i)) and CV (B(ii)) variations at location **a** (marked in A) dependent on simulated beats. C. Spatial distributions for APD (C(i)) and CV (C(ii)) for beat 1,2,3,13,14. D. Time course traces for location **a**, **b**, **c**, **d**, **e** marked in A.

AP alternans at the cellular level might be mapped into spatio-temporally heterogeneous conduction, resulting in functional heterogeneity leading to impaired excitation wave conduction at 1D tissue level. Fig 3 shows results of simulated excitation wave conduction paced at PCL = 140 ms in a 1D homogenous strand model in control condition (*S*_*gNa*_ = 1.0). In the figure, the evoked action potential propagation along the strand was colour mapped and plotted in the space-time domain, in which space goes vertically from the bottom to the top, and time goes horizontally from the left to the right (Fig 3A). It was shown at the local region of the stimulation site, the stimuli evoked a series of APs, with a large one being followed by a small one, showing apparent electrical AP alternans. Correspondingly, a fast wave followed by a slow one was observed initially at the vicinity of the stimulation site (Fig 2A(i)), which was mapped into distant tissue regions later on.

To illustrate electrical and conduction alternans, time courses of the computed APD and CV near the stimulus region (a red line marked by **a** in Fig 3A) were plotted simultaneously in Fig 3B(i-ii). Distinctive oscillating AP and CV were observed from the 6^th^ beat, with a larger APD being correlated with a greater CV, and vice versa. With a small AP, a slow CV at the vicinity of the stimulation site was observed, resulting in a conduction delay, allowing other part of the tissue more time to recover from a previous excitation. When the small AP excitation wave reaches the more recovered part of tissue, it became large and conducted relatively quickly until the excitation wave reaches the refractory tail of the previous excitation. In such a way, a profound functional spatial heterogeneity was generated in the intrinsically homogeneous tissue. With time, the spatial heterogeneity evolves into a large scale spatial-temporal heterogeneity, by which standing waves were observed. In this case, along the strand, regions of larger AP were alternated by those of smaller AP, each of which was associated with a faster or a slower conduction velocity accordingly.

Evolution of the functional spatial-temporal heterogeneity along the strand was characterised by the spatial distribution of the APD and CV from beat to beat as illustrated in Fig 3C(i-ii). At beat 1, both APD and CV showed almost a homogeneous spatial-distribution (a slight inhomogeneous distribution of APD and CV along the strand was due to the boundary effect of the tissue model [51]). Following a premature beat that failed to evoke propagation, the spatial heterogeneity was built up and became noticeable at beat 4, a smaller APD and CV was observed at the region near the stimulation site as compared to those at distance. With time, a significant spatial heterogeneity with respect to both APD and CV through the whole tissue strand was produced and became markedly at beat 14 and beat 15, leading to the formation of standing waves.

With the formation of standing waves, electrical alternans in the tissue showed both concordant and discordant features, depending on the spatial scale of observation. Fig 3D shows the time course of AP traces recorded from position ***a*** to position ***e*** (marked in Fig 3A). APs registered from different sites of the strand showed either concordant or discordant alternans. Within a small observation scale (within (***a***, ***b***, ***c***) or (***d***, ***e***)), recorded APs (from ***a*** to ***c***: marked by red arrows; from ***d*** to ***e***: marked by blue arrows) showed clearly concordant alternans. Within a large observation scale (between (***a***, ***b***, ***c***) and (***d***, ***e***)), APs showed significantly discordant alternans. Note that there is a singularity point, by which in-phase and out-of-phase APs were separated. As such, APs recorded from ***c*** and ***d***, though the distance between them was small, clearly showed discordant alternans.

Correlation between alternating APD and alternating CV was also observed in the CRN model of the homogeneous human atrial strand, which generated the functional heterogeneity leading to formation of standing waves as shown in S3.2 Fig in the Online Supplement.

**Fig 4:**
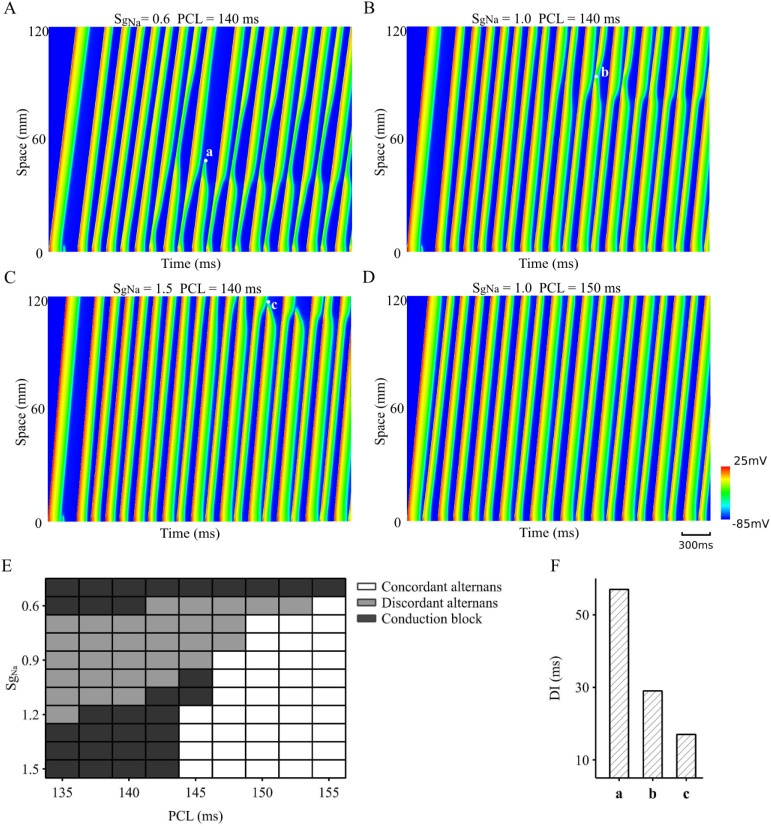
Role of *I*_*Na*_ in alternans conduction in the 1D tissue model. A. Reduced *I*_*Na*_ (*S*_*gNa*_ = 0.6) leading to discordant alternans and conduction block with PCL = 140ms. B. Control *I*_*Na*_ (*S*_*gNa*_ = 1.0) leading to discordant alternans with PCL = 140ms; C. Increased *I*_*Na*_ (*S*_*gNa*_ = 1.5) leading to discordant alternans and conduction block with PCL = 140ms. D. Concordant alternans generated in control *I*_*Na*_ (*S*_*gNa*_ = 1.0) with PCL = 150ms. E. Map of genesis of concordant, discordant and conduction block in the PCL-*S*_*gNa*_ parameter space. F. Computed DI between two consecutive excitation waves at the marked beats and locations for generation of conduction break in (A-C).

The role of *I*_*Na*_ in the conduction of AP alternans was further investigated in the 1D tissue model. Results are shown in Fig 4 for the space-time plot of AP conduction with a reduced (Fig 4A), control (Fig 4B) and an increased *I*_*Na*_ (Fig 4C). At PCL = 140ms, a reduced *I*_*Na*_ facilitated the transition from a standing wave to a conduction block (Fig 4A) at some of the singularity points. Similarly, an increased *I*_*Na*_ also led to a conduction failure of the standing waves (Fig 4C), though with a greater distance from the stimulation site as compared to the case of *I*_*Na*_ reduction. These results suggested that *I*_*Na*_ plays an important role in the conduction of AP alternans: either reducing or increasing it may result in the transition from discordant alternans to conduction failure. The genesis of discordant alternans was also PCL-dependent, as shown in Fig 4B and 4D. By increasing PCL from 140 ms to 150 ms, the observed discordant alternans became concordant alternans in the tissue.

A complete map in the 2D PCL-*S*_*gNa*_ parameter space was constructed to demonstrate the combined effects of altered PCL and *S*_*gNa*_ on the genesis of concordant, discordant alternans and the generation of conduction failure. Results are shown in Fig 4E. In the figure, white blocks represented the area within which only concordant alternans appeared, grey blocks for the area of discordant alternans with conducting standing waves, and black blocks for the discordant alternans with conduction failure. It was shown that when *S*_*gNa*_ was smaller than 0.6, a conduction block always occurred either at the vicinity of the stimulus site or in the middle of the strand for a wide range of PCLs. This suggested a decreased *I*_*Na*_ might facilitate the genesis of discordant alternans and conduction failure at low heart rate, resulting in an increased risk of arrhythmogenesis.

On the other hand, an increased *I*_*Na*_ also led to discordant alternans and conduction failure at fast pacing rates (*i*.*e*., small PCLs), but not at slow pacing rates. This is paradoxical in light of the prediction of single cell simulations which showed an increased maximum slope of APD and CV restitution curves with an increased *I*_*Na*_ (Fig 1I and Fig 2B(ii)), by which one would expect a more pronounced genesis of alternans. Such a discrepancy between cell and tissue modelling may be attributed to different mechanisms by which a reduced or increased *I*_*Na*_ facilitates alternans conduction. In the case with a reduced *I*_*Na*_, the reduction in the upstroke velocity of the small AP resulted in a remarkable slow CV during propagations. In the case when AP was small enough to reach the threshold for propagation, conduction failure occurred. For an increased *I*_Na_, the difference between large and small APs became more pronounced, leading to a large gradient of APD dispersion in the tissue, which finally caused conduction block. To test this hypothesis, the diastolic interval (DI) between the failing wave and the previous one at the conduction block sites was measured (marked in Fig 4A–4C as ***a***, ***b***, ***c***) and presented in Fig 4F. It was shown that the computed DI with a reduced *I*_*Na*_ was significantly greater than that in control and increased *I*_*Na*_ condition, suggesting the conduction failure for an increased *I*_*Na*_ was due to a reduced DI, by which the excitation wave collided with the tail of the previous excitation, leading to self-termination. However, with a reduced *I*_*Na*_, a large DI suggested that the conduction failure was attributable to a small AP that failed to provoke excitation.

Effects of varied recovery time of *I*_*Na*_ on alternans genesis and conduction was investigated, and results are shown in S4 Fig in the Online Supplement for the 1D tissue model with a reduced (*S*_*gNa*_ = 0.6), normal (*S*_*gNa*_ = 1.0), and increased (*S*_*gNa*_ = 1.5) *I*_*Na*_ conditions at different PCLs. For each condition, the distance between the stimulation site and the first APD node, *i*.*e*., the point where distinctive discordant alternans first appeared was computed (see the black lines marked in S4A(i-ii) Fig) Fig to measure a minimal tissue size required for developing and sustaining discordant alternans. It was shown that a shortened APD node distance was associated with a longer *I*_*Na*_ recovery time, suggesting a facilitative role of impaired *I*_*Na*_ recovery in generating discordant alternans.

### Discordant alternans in 2D simulations

**Fig 5:**
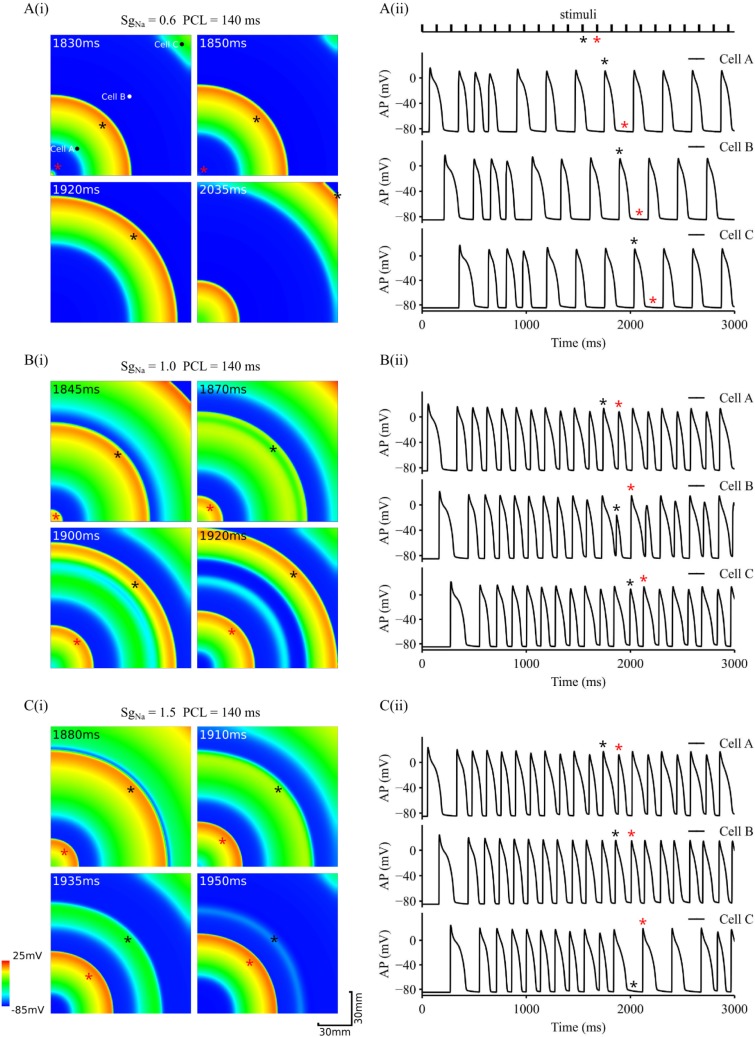
Snapshots of conduction of AP alternans in 2D tissue and time series of APs. APs were recorded from three different registration sites (cell A, cell B and cell C marked at the tissue shown in the top-left panel of A(i)). PCL = 140ms. A(i-ii). Tissue with reduced *I*_*Na*_ (*S*_*gNa*_ = 0.6). B(i-ii). Control tissue (*S*_*gNa*_ = 1.0). C(i-ii). Tissue with increased *I*_*Na*_ (*S*_*gNa*_ = 1.5). Black and red stars marked two consecutive stimulus pulses as shown in the top-panel of A(ii), and corresponding excitation waves in the tissue (panels in A(i) and B(i) and C(i)), as well as APs recorded from the three registration sites (A(ii) and B(ii) and C(ii)).

Excitation waves in cardiac tissue resemble curved waves more than planar waves as seen in the 1D tissue. Therefore, further simulations were conducted to investigate the conduction of curved excitation waves associated with alternans in a homogeneous 2D tissue model. Effects of a decreased and increased *I*_*Na*_ on the conduction of the excitation waves were also investigated. Results are shown in Fig 5.

Fig 5A shows snapshots of excitation waves in the 2D tissue with a reduced *I*_*Na*_ (*S*_*gNa*_ = 0.6). These excitation waves were evoked by a series of stimuli applied at the left bottom corner with PCL = 140ms (Fig 5A(i)). With such a PCL, AP alternans and discordant alternans were observed at the cellular and 1D tissue levels respectively. However, in the 2D tissue model, due to the combined effects of wave curvature and electronic interactions between cells, 1:1 alternans seen at the 1D tissue disappeared. Instead, intermittent excitations switching between fast and slow rates were observed as indicated by the time series of action potentials (Fig 5A(ii)) recorded from registration sites of cell A, cell B and cell C (sites marked in Fig 5A(i)). Interestingly, some beats of the stimuli failed to evoke excitation wave propagation in the tissue. For example, for the 12th and 13th stimulus beats marked as black and red star respectively in the stimuli time series as shown in Fig 5A(ii), one generated excitation propagation (12^th^ beat), and the other failed. In this way, 1:1 response of the tissue to stimuli failed. Conduction failure gave tissue enough time to recover from the previous excitation, leading to a normal conduction across the 2D tissue with no alternations. However, the excitation rate was halved.

For the control condition (*S*_*gNa*_ = 1.0), by which standing waves were observed in the 1D strand model, functional heterogeneity was generated in the 2D tissue model (Fig 5B(i)). In this case, the small AP of the alternans conducted slowly at some local regions (marked by black star of the wavefront in Fig 5B(i)), allowing the other parts of the tissue more time to recover from the previous excitation. When the excitation wave reached the regions with more recovered excitability, it conducted relatively faster until it hit the refractory tail of the previous excitation, where the conduction became slowed down again or even stopped. This led to the formation of excitation-refractory islands, generating spatial functional heterogeneity in the tissue, which became more pronounced with time. For example, at the 12^th^ beat of stimulus with wave front marked by the black star in Fig 5B(i), the wave front caught up the rear of the wave of the previous excitation, resulting in a small AP (therefore small CV) at recording site of cell B (Fig 5B(ii)), which evolved into a repolarisation island (a green strip after the wave front marked by the black star in Fig 5B(i)). In this case, discordant alternans was observed as shown in the time series of recorded APs (Fig 5B(ii); see black stars and red stars).

With an increased *I*_Na_, AP propagation in the 2D tissue model was similar to those observed in 1D model. In addition to discordant alternans, conduction might fail when the wave front hit the refractory tail of the previous AP excitation as shown in Fig 5C(i). For example, at the 12^th^ beat of stimuli, the excitation wavefront (marked by the black star) collided with its processor, leading to terminated propagation (Fig 5C(i)). This formed a conduction block zone to the wave front of the next excitation (i.e., evoked by the 13^th^ stimulus; marked by the red star), leading to conduction failure. APs recorded from registration sites clearly showed missing beats at the distant site (cell C) from the stimulus region. In such a way, tissue failed to respond 1:1 to the stimuli.

### Transition from discordant alternans to re-entrant arrhythmia

In the following simulations, we investigated the transition from conduction alternans to re-entrant arrhythmias in homogeneous, inhomogeneous and anisotropic 2D tissue models in order to understand possible roles of tissue inhomogeneity and anisotropy in perpetuating the formation of re-entrant excitation. Due to conduction failure in 2D tissue with reduced *I*_Na_, simulations results with control (*S*_*gNa*_ = 1.0) and increased *I*_*Na*_ (*S*_*gNa*_ = 1.5) at PCL = 140 ms were shown in the following sections.

**Fig 6:**
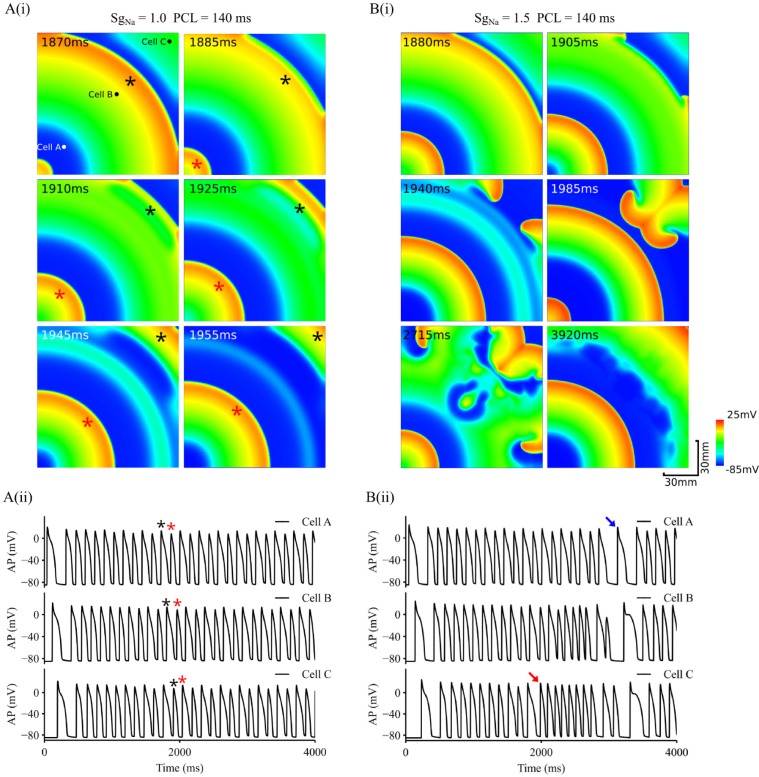
Role of *I*_*Na*_ in transition from discordant alternans to re-entrant arrhythmias. A(i). Snapshots of excitation waves in a homogeneous tissue with control *I*_*Na*_. PCL = 140ms. A(ii). Time series of APs recorded from 3 different registration sites, cell A, B and C as marked by black dots in the top-left panel. B(i). Snapshots of excitation waves in a homogeneous tissue with increased *I*_*Na*_. PCL = 140ms. B(ii). Time series of APs recorded from 3 different registration sites, cell A, B and C as marked by black dots in the top-left panel of A(i). Red arrow: marking for beginning of re-entry. Blue arrow: marking for termination of re-entry. Black and red stars marked two consecutive stimulus pulses. Simulations presented here were done with D = 0.18 mm^2^/ms.

In the 2D homogeneous tissue model with increased *I*_Na_ (S_gNa_ = 1.5), alternating excitation wave conduction might transit spontaneously to paroxysmal re-entrant excitation waves when conduction failure associated with the small AP occurred near the tissue boundary as shown in Fig 6. In simulations, condition and parameters of the model were the same as used in Fig 5, but with a slight increase in the conduction velocity (by about 10%), allowing conduction slowing down to occur near the tissue boundary. With normal *I*_Na_ (S_gNa_ = 1.0), though conduction slowed down when the excitation wavefront ran into the refractory tail of the previous excitation (snapshot at 1885 ms), it continued to propagate without breakup due to sufficient gap between the two consecutive waves. However, with S_gNa_ = 1.5 (Fig 6B(i)), after a transition period of 1880 ms during which functional heterogeneity developed in the tissue, the wavefront of the excitation wave (*i*.*e*., the one in the middle of the tissue) collided with the refractory tail of the previous excitation, leaving a very narrow refractory gap between the two due to more pronounced AP alternans as compared to the case of S_gNa_ = 1.0. In this case, the conduction of wavefront slowed down, or stopped especially in the middle parts with a greater curvature. However, at the tissue boundaries, as the Neumann non-flux boundary condition was implemented, effective intercellular coupling of cells was slightly less loaded as compared to other parts of tissue, leading to a slightly increased excitability of the tissue. Consequently, the excitation wave conducted preferentially along the boundaries, leading to formation of two new small daughter waves with a smaller curvature as compared to the mother excitation. A combined action of higher cellular excitability and less wave curvature in the tissue allowed the two daughter waves to conduct faster into the region recovered from the previous excitation, leading to the formation of the re-entry. APs recorded from different registration sites in the tissue showed out-of-phase excitation (Fig 6B(ii)) before the onset of re-entry (marked by the red arrow), which self-terminated after a short period of 1 s (marked by the blue arrow), afterwards discordant alternating excitation resumed with a more marked functional heterogeneity in the tissue (see distorted wave back at 3920ms in Fig 6B(i)). Note that boundaries arising from non-excitable valve annuals or fibrosis boarders exist in cardiac tissue, around where arrhythmia origins are frequently located [55,56].

**Fig 7:**
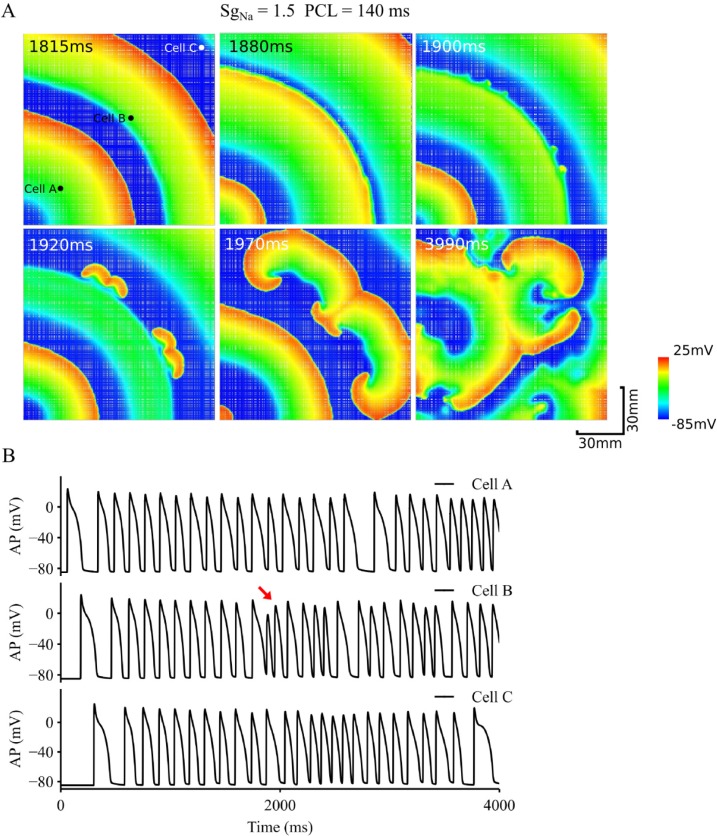
Role of inhomogeneity in transition from discordant alternans to re-entrant arrhythmias. A. Snapshots of excitation waves in an inhomogeneous tissue with increased *I_Na_*. PCL = 140 ms. B. Time series of APs recorded from 3 different registration sites, cell A, B and C as marked by black dots in the top-left panel. Red arrow: marking for the beginning of re-entry. Inhomogeneous tissue was simulated by assigning cells to dead cells randomly in the tissue.

In the inhomogeneous tissue model, inclusion of 10% of dead cells (illustrated by white nodes in Fig 7A) caused distorted wavefronts due to non-uniform conduction velocity in the tissue. At places where wave curvature was pronounced and dead cell population was high, the wavefront of the excitation wave associated with the small AP alternans broke down, leading to formation of re-entry, which sustained through the whole period of simulation (4s) as shown in Fig 7B. This re-entry further broke down leading to multiple wavelets, which is one feature of fibrillation.

Further simulations were also conducted to dissect the relative role of tissue inhomogeneity in promoting the transition from alternans to re-entry. Results shown in S5.1 Fig in the Online Supplement were obtained from an inhomogeneous 2D tissue model (inhomogeneity was simulated by assigning 10% of cell population as dead cells illustrated by white nodes in S5.1A Fig) with *S*_*gNa*_ = 1. In the model, wave-break occurred, leading to the formation of sustained re-entries. This was different to the results shown in Fig 5B(i) for a homogeneous model with *S*_*gNa*_ = 1, where though functional heterogeneity manifested as small excitation-refractory islands were observed, but no re-entry was initiated. Therefore, in the tissue model without impaired *I*_*Na*_, inhomogeneity plays an important role in promoting the spontaneous transition from alternans to arrhythmias.

**Fig 8:**
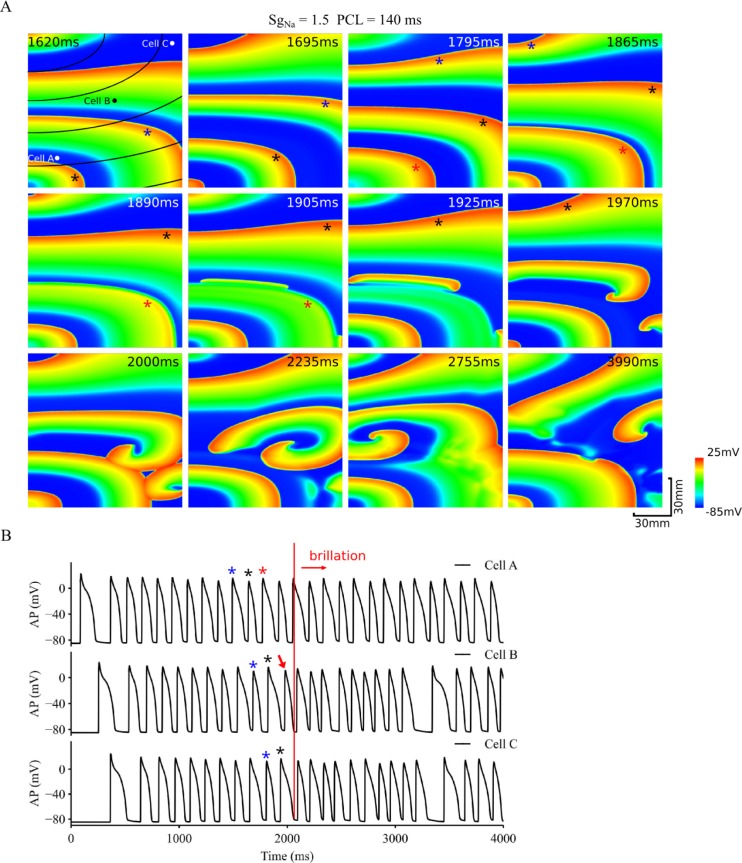
Role of anisotropy in transition from discordant alternans to re-entrant arrhythmias. A. Snapshots of excitation waves in a homogeneous but anisotropic tissue with increased *I*_*Na*_. PCL = 140 ms. B. Time series of APs recorded from 3 different registration sites, cell A, B and C as marked by black dots in the top-left panel. Red arrow: marking for the beginning of re-entry; Blue, black and red stars marked three consecutive stimulus pulses from the 10^th^ stimulus.

The role of anisotropy of the tissue in facilitating the transition from discordant alternans to re-entry is shown in Fig 8, which shows snapshots of alternating excitation waves in the tissue model with a 2:1 ratio of CV along and perpendicular to the fibre (fibres are shown by black lines in the top-left panel of Fig 8A). Arising from anisotropic propagation, excitation wavefront became non circular (i.e., unsymmetrical) with a non-uniform curvature. Similar to the isotropic tissue, the functional heterogeneity generated by alternans conduction also developed, leading to formation of standing wave conduction (e.g. the excitation wave marked by the blue star altered from a large AP excitation to a small one during its conduction course as shown by the snapshots at 1620 ms and 1695 ms in Fig 8A). Such functional heterogeneity evolved with time, and eventually caused the wave front (marked by the red star) to collide with the wave back of the previous wave (marked by the black star) (see snapshot at 1865ms in Fig 8A), leading to a slow conduction or even a paused conduction at places with greater wavefront curvature (see snapshots at 1890 and 1905 ms). However, at places where the curvature was small and the conduction direction was more in parallel with the fibre direction, conduction continued due to a less curvature impact on CV of curved excitation [57], leading to formation of new daughter wavelets, which evolved into re-entrant excitation waves (see snapshots from 1905 to 1970 ms) that further broke down into multiple wavelets (see snapshots from 1970 to 3990 ms).

Once initiated, re-entry sustained through the whole period of 4s simulation as shown in Fig 8B, in which recorded AP time courses from three registration sites (marked as cell A, B and C in the top-left panel of Fig 8A) are shown. In the figure, black (blue) stars mark the transition from a large (small) AP at place A to a small (big) AP at place B and C, indicating the features of discordant and standing wave conduction. At the stimulus pulse marked by the red star, a large AP was recorded at place A, but it became a small one at location B, and was missing at location C. The tissue around C was re-excited when re-entry excitation formed at the timing marked by the red arrow.

However, in the anisotropic model with *S*_*gNa*_ = 1, there was no wave break observed to generate the spontaneous transition from alternans to re-entry as shown in S5.2 Fig, also highlighting the important role of *I*_*Na*_ in promoting such a transition.

### Spontaneous transition from alternans to re-entry in a 2D human atrial model

Further simulations were conducted in a 2D model of the human atrium to test if discordant alternans can spontaneously transit into re-entry. Results shown in S3.3 and S3.4 Fig in the Online Supplement were obtained from the model with homogeneous and anisotropic tissue properties respectively. As shown in S3.3 Fig, in the homogeneous model standing waves were observed producing functional heterogeneity, but there was no wave break observed, which is a similar to the results as shown in Fig 5 for the rabbit ventricle tissue model. However, in the model with anisotropic AP conduction as shown in S3.4 Fig, the wave front became unsymmetrical, leading to the development of functional heterogeneity, which eventually evolved into spontaneously generated re-entrant excitation waves.

## Discussion

In this study, we investigated the mechanisms by which AP alternans at the cellular level evolves into discordant alternans at the tissue level, which further evolves into re-entrant excitation waves. The role of a reduced *I*_*Na*_ or increased *I*_*Na*_ on facilitating the transition from AP alternans to discordant alternans was investigated. Effects of tissue inhomogeneity and anisotropy on perpetuating the transition from discordant alternans to re-entrant arrhythmias were also investigated. Our major findings are: (i) the combined action of rate-dependent APD and CV restitution properties underlie the genesis of discordant alternans at the tissue level, leading to formation of standing waves; (ii) both concordant and discordant alternans can be observed in the same tissue depending on the observation spatial scale, and there is a singularity point separating the concordant region from the discordant region; (iii) conduction of discordant alternans produces functional heterogeneity in the homogeneous tissue, which evolves with time causing conduction block; (iv) altered *I*_*Na*_ (either reduced or increased) modulates tissue’s conduction restitution property, leading to either conduction failure or conduction block of the alternans, forming a substrate favourable for arrhythmogenesis; (v) in 2D tissue, the combined action of CV restitution and curvature-dependence of wave front conduction breaks up the wavefront at some local points, leading to formation of re-entrant excitations; (vi) inhomogeneity or anisotropy of the tissue promotes the wavefront to break up, leading to sustained re-entrant excitation wavelets–a condition seen in cardiac fibrillation. These results add more details to support previous findings [29] on how AP alternans transits into discordant alternans, and most importantly provide new mechanistic insights for understanding the spontaneous transition from AP alternans to cardiac fibrillation, especially the role of *I*_*Na*_, tissue inhomogeneity and anisotropy. These major findings are also observed in human atrial cell and tissue models, suggesting them apply across different models and to have human relevance.

### Mechanisms of transition from AP alternans to discordant alternans and then to re-entry

Spatially discordant alternans manifested by out-of-phase excitation between two neighbouring regions is believed to facilitate the formation of re-entry, underlying cardiac fibrillation [24]. In this study, we demonstrated that spatially discordant alternans arose from the combined action of APD restitution and CV restitution properties of cardiac tissue. In response to a sequence of fast pacing, AP alternans was generated due to APD restitution property (Fig 1A). Correspondingly, CV alternans was produced due to its restitution property, resulting in a large AP associated with a fast CV and vice versa (Fig 3B). In the case when AP alternans was not sufficient to generate marked CV alternans, APD and CV remained stable and uniform across the tissue, then concordant alternans was generated (Fig 4D). However, in the case when AP alternans were associated with marked CV alternans, then the slower CV associated with the smaller APD delayed the conduction, leaving more recovery time for the tissue region ahead of the wavefront, resulting in a larger AP and faster CV when the wavefront reached the region (see Fig 3 and Fig 4). Consequently, conduction of standing waves was generated, which was associated with a functional heterogeneity. With time, such a functional heterogeneity in the homogeneous tissue became more significant, facilitating more marked discordant alternans (Fig 3A).

Associated with standing waves, previous theoretical analyses [29] have predicted a location of the APD node (i.e., a singularity point) in the tissue, which marks the division of two out-of-phase regions, whilst the APD at the nodal point remains constant and stable with time. In our simulations using biophysically detailed model of cardiac tissue, such a APD nodal point was also observed with small APD variations (the site between location **c** and **d** in Fig 3A) and drifting with time. Within both sides of the nodal point, concordant alternans was observed; whilst between the two sides, discordant alternans appeared (Fig 3D). Such a spatial scale-dependent observation of concordance and discordance may explain why concordant alternans were observed in some studies [58,59] and discordant alternans were observed in some other studies [24,60].

The transition from discordant alternans to re-entrant excitation waves arises from a combined action of cardiac restitution properties (APD and CV) and the curvature effect on the conduction velocity of the wavefront. It has been shown that the curvature modulates the conduction velocity of a curved wavefront: the more concaved the curvature, the slower the conduction speed [57]. In the 2D tissue models, the evoked excitation waves first evolved into standing waves with functional spatial heterogeneity. Such a functional heterogeneity either led to conduction failure or repolarisation islands that hampered wave conduction. In the case when conduction terminated, in some localised regions of the wavefront where there was a smaller curvature or greater cell excitability, either due to the intrinsic features of the tissue or arising from boundary effects, conduction survived, leading to the development of new daughter waves that evolved into re-entry. Note that abundant boundaries exist in cardiac tissue due to non-excitable blood vessels and valve annuals, which form major source of arrhythmia origins [55,61].

Cardiac tissue is inhomogeneous and anisotropic. Heterogeneity may arise due to intrinsic differences in cellular properties, or to the presence of populations of dead cells and fibrosis. Our simulations showed that such inhomogeneity in cardiac excitability and tissue anisotropy play an important role to facilitate the breakup of the alternating wavefront, leading to sustained re-entrant excitation waves. This occurred through the combined action of discordant alternans, anisotropic conduction and distorted wavefront/waveback, which causes the breakup of the excitation wavefront, forming re-entry.

### Role of *I*_*Na*_ on discordant alternans and conduction block

*I*_*Na*_ drives the AP upstroke and therefore affects AP conduction in the cardiac tissue [42]. Malfunctions of channels underlying *I*_*Na*_ have been identified in various conditions, including the sick sinus syndrome (SSS) [62] and conduction disease [40,63]. Remodelling of *I*_*Na*_ channel has also been identified in heart failure [64] and cardiac ischaemia [65]. Genetic defects of *I*_*Na*_ channel subunits have also been identified in some inherited diseases, with loss-of-function mutations being associated with SSS [66] and Brugada Syndrome [67], and gain-of-function mutations with long QT syndrome [68]. Studies have also found that malfunction of *I*_*Na*_ modulates the effective refractory period between cardiac action potentials, and thereby to contribute to the genesis of cellular alternans [42,69]. Thus, it is of interesting to underpin the role of *I*_*Na*_ in perpetuating the genesis of discordant alternans leading to formation of re-entry.

In this study, it was found at the cell level that an increased *I*_*Na*_ resulted in a greater difference of APD and *dV/dt*_max_ between large and small APs (Fig 1D–1F), and to a steeper APD restitution curve (Fig 1H–1I). At the tissue level, it produced a greater difference between fast and slow CV associated with large and small APs respectively (Fig 2A), as well as an increased steepness of the CV restitution curves (Fig 2B). Such an increased difference in APD and CV between big and small APs, and the steepness of their restitution properties resulted in greater functional heterogeneity, leading to increased risks of conduction block and re-entrant excitation. With an increased *I*_*Na*_, the critical PCL value required for alternans was reduced (Fig 4E) (i.e., higher heart rates); however once formed, discordant alternans easily produced conduction block or formation of re-entry.

As compared to the control condition, a reduced *I*_*Na*_ decreased the difference between large and small APs, but increased the ERP (Fig 1G). In addition, it increased the critical PCL value (i.e., a reduced heart rate) for generating alternans, implying a greater role in predisposing towards generation of discordant alternans. The increased ERP caused conduction failure in both 1D and 2D simulations.

Our results show that impaired *I*_*Na*_ plays an important role in perpetuating the transition from discordant alternans to re-entry by an integral action with other factors. This can be illustrated by comparing results obtained from 2D tissue models without altering the channel conductance (i.e., *S*_*gNa*_ = 1), but considering homogeneous (Fig 6A), inhomogeneous (S5.1 Fig) or anisotropic tissue (S5.2 Fig) properties respectively. In the homogeneous (Fig 6A) or anisotropic (S5.2 Fig) model of the rabbit ventricle tissue, though obvious standing waves were observed, which evolved into functional heterogeneity with repolarisation islands, no re-entry was observed. Only in the model with inhomogeneous tissue properties did wave-break occur, leading to the formation of re-entry. This is different to the case when impaired *I*_*Na*_ was considered as shown in Figs 6B, 7 and 8, in which a spontaneous transition was observed for homogeneous (Fig 6B), inhomogeneous (Fig 7) and anisotropic (Fig 8) tissue models with *S*_*gNa*_ = 1.5. Therefore, in the tissue model without impaired *I*_*Na*_, tissue inhomogeneity may promote the transition from alternans to arrhythmias; but in tissue with impaired *I*_*Na*_, such a transition can occur in absence of tissue inhomogeneity or anisotropy.

Prolonged *I*_*Na*_ recovery time may also play a role for facilitating the transition from alternans to re-entry. With varied levels of the increase in the time constant of the inactivation, discordant alternans was observed with the measured APD node distance being decreasing with the increase of the recovery time, suggesting greater susceptibility of the tissue to genesis and accommodation of discordant alternans, as the smaller the APD node distance the smaller of the tissue size is required to accommodate the discordant alternans.

### Limitations and future work

The limitations inherited from the Aslanidi et al. model have been discussed elsewhere [43,70]. In this study, in order to investigate mechanisms responsible for the transition from alternans to arrhythmia, we used a large tissue size (120mm by 120mm). Such a tissue size is larger than the size of rabbit heart. However, considering the 3D spatially complex geometry, especially the large area of heart surface, such tissue size may be reasonable to demonstrate the full evolution of spatial heterogeneity in association with discordant alternans. In simulations, we noted that the wavefront broke-up at about the centre of the tissue model, which reduced the effective size for re-entry initiation to about half of the size used. We also noted that with the increase of the *I*_*Na*_ recovery time, the size required for induction of wave-break further reduced, allowing the tissue size to be more comparable to the surface area of the ventricle walls. In the model, inhomogeneity and anisotropy of the tissue were considered, but they were putative. Future studies based on 3D anatomical geometry, detailed cellular heterogeneity and realistic tissue anisotropy are warranted. Also, it is known that TWA is mainly associated with abnormal repolarisation process of the action potential [36], during which some repolarisation potassium channel currents play important role [34,35]. Effects of repolarising potassium channel current on modulating the transition from alternans to spontaneous transition were not considered here, but are of interest, warranting future studies.

While it is helpful to identify these limitations, our major conclusion on the role of tissue heterogeneity and anisotropy remains the same.

### Relevance of the study

Possible mechanisms responsible for the genesis of cardiac alternans in electrical action potentials and/or in the intracellular calcium transients have been extensively studied in previous experimental [18,19,71] and numerical simulation studies[14,72,73]. Roles of cardiac alternans in arrhythmogenesis have also been investigated to show how it augments tissue functional heterogeneity that causes conduction block [74–76], leading to arrhythmogenesis due to the combined action of pre-existing tissue intrinsic heterogeneity in cellular electrophysiology, intercellular coupling and/or anisotropy [30,77–79]; or in the presence of additional factors of PVCs, DADs or EADs [80–83]. Effects of wavefront curvature on the stability of re-entrant excitation wave have also been investigated in previous studies [84,85]. However, to our best knowledge a spontaneous transition from discordant alternans to initiation of re-entry due to an integral action of tissue functional heterogeneity, APD and CV restitution properties and the curvature-dependence of excitation wavefront has not been demonstrated before.

Impaired sodium channel function plays an important role in the genesis of cellular alternans and promoting discordant alternans in tissue models. Qu et al. [86] have previously shown that reduction in the Na^+^ channel conductance or slowing its recovery prolongs ERP, alters APD restitution, reduces CV, and broadens CV restitution, promoting discordant APD alternans. It may also cause cellular APA alternans that is linked to QRS alternans [87], contributing to initiation of spiral waves and complex dynamics of re-entrant excitations. Whilst our simulation results are consistent with those findings in demonstrating the role of a reduced *I*_*Na*_ in promoting alternans genesis, we also provide new findings suggesting that an abnormally augmented Na^+^ channel conductance also promotes alternans.

## Conclusions

This study provides new insights into the understanding of cardiac arrhythmogenesis in association with cardiac alternans, an important clinical observation. Possible mechanisms by which AP alternans evolve into discordant alternans and then re-entrant excitation waves have been determined. Simulation results from human atrial models suggest that the major findings of the present study are model-independent and have potential clinical relevance.

## Supporting information

S1 Text Supplementary materials for methods and results. (PDF)

S1 Fig S3.1 Fig Cellular model properties of CRN model. (PDF)

S2 Fig S3.2 Fig Representative 1D simulation results at PCL = 320ms. (PDF)

S3 Fig S3.3 Fig Snapshots of conduction of AP alternans in 2D homogenous tissue and time series of APs. (PDF)

S4 Fig S3.4 Fig Snapshots of conduction of AP alternans in 2D anisotropy tissue and time series of APs. (PDF)

S5 Fig S4 Fig Role of *I*_*Na*_ recovery time in the location of APD node. (PDF)

S6 Fig S5.1 Fig Conduction of AP alternans in 2D inhomogeneous tissue with normal *I*_*Na*_ (S_gNa_ = 1) and time series of APs. (PDF)

S7 Fig S5.2 Fig Conduction of AP alternans in 2D anisotropic tissue with normal I_Na_ and time series of APs. (PDF)
