# Supplementary material for: Mechanistic insight into spontaneous transition from cellular alternans to arrhythmia—A simulation study
Source: PLoS Comput Biol. 2018 Nov 30;14(11):e1006594. doi: 10.1371/journal.pcbi.1006594 (PMC6291170; doi:10.1371/journal.pcbi.1006594)
Supplement: S1 Text — (PDF) [file pcbi.1006594.s001.pdf]

Supplementary materials for methods and results

S1. Equations for Fast Na^+ current

$$I_{Na} = S_{g_{Na}} g_{Na} m^3 hj (V_m - E_{Na})$$

$$E_{Na} = \frac{RT}{F} \ln \frac{[Na]_e}{[Na]_i}$$

$$\frac{dm}{dt} = \frac{m_\infty - m}{\tau_m}$$

$$m_\infty = \frac{\alpha_m}{(\alpha_m + \beta_m)}$$

$$\tau_m = \frac{1.0}{(\alpha_m + \beta_m)}$$

$$\alpha_m = \frac{0.32(V + 47.13)}{1 - e^{-0.1(V + 47.13)}}$$

$$\beta_m = 0.08 e^{-V/11}$$

$$\frac{dh}{dt} = \frac{h_\infty - h}{\tau_h}$$

$$\frac{dj}{dt} = \frac{j_\infty - j}{\tau_j}$$

$$h_\infty = \frac{\alpha_h}{(\alpha_h + \beta_h)}$$

$$j_\infty = \frac{\alpha_j}{(\alpha_j + \beta_j)}$$

$$\tau_h = \frac{1.0}{(\alpha_h + \beta_h)}$$

$$\tau_j = \frac{1.0}{(\alpha_j + \beta_j)}$$

If V ≥ -40mV:

$$\alpha_h = 0$$

$$\beta_h = \frac{1}{0.13(1+e^{(V+10.66)/-11.1})}$$

$$\alpha_j = 0$$

$$\beta_j = \frac{0.3e^{-2.535\times10^{-7}V}}{1+e^{-0.1(V+32)}}$$

If V < -40mV:

$$\alpha_h = 0.135e^{(80+V)/-6.8}$$

$$\beta_h = 3.56e^{0.079V} + 3.1\times10^5 e^{0.35V}$$

$$\alpha_j = \frac{-1.2714\times10^5 e^{0.2444V} - 3.474\times10^{-5} e^{-0.0439V} \times (V+37.78)}{1+e^{0.311(V+79.23)}}$$

$$\beta_j = \frac{0.1212e^{-0.01052V}}{1+e^{-0.1378(V+40.14)}}$$

If V < -70mV:

$$\tau_h = \tau_h + 30$$

S2. Parameters for Fast Na^+ current

Parameter	Value	Units
g_{Na}	16	mS/µF
$[Na]_e$	145	mM
T	37	˚C

S3. Model-independence - results from a human atrial model.

To test if our main findings are model dependent, simulations were repeated in a human atrial model published by Courtemanche et al. (denoted as CRN model, [1]), which is able to generate stable AP alternans. The model was chosen to demonstrate that the main mechanisms of the spontaneous transition from cellular alternans to arrhythmia is not only model-independent, but also species-independent. For the CRN model simulations, the same simulation protocols and numerical methods as used in the main manuscript were used. The model was paced 10 beats with PCL of 1000ms to give the initial conditions to all following simulations. For 1D simulations, a strand of a total length of 90mm was discretised by a spatial resolution of 0.3 mm to form 300 interconnected nodes, each of which was modelled by the CRN cell model, with the diffusion coefficient D resetting to 0. 05mm^2/ms to give a conduction velocity of about 0.3m/s for human atria. For 2D simulations, an idealised geometry of cardiac tissue sheet with 90×90 mm^2 were used. In isotropic tissue models, the diffusion coefficient D was set to be the same value as that used in the 1D simulation. In anisotropic tissue models, D for the direction

in parallel to the fibre direction remains the same as that in the 1D, and for the direction perpendicular to the fibre was set be a quarter of that along the fibre, which gave a 2:1 ratio of the CV for along and perpendicular to the fibre. The fibre orientation was implemented using previous Equation (5).

Fig S3.1 shows a representative AP alternans generated by the CRN model at PCL = 320ms, in which obvious large and small AP alternans (Fig S3.1 A and B) was observed. Also, this AP alternans is associated with large and small sodium (Fig S3.1 C) and L-type calcium (Fig S3.1 D) currents. Fig S3.1E and F showed a steep the APD restitution curve and CV restitution curve of the CRN model calculated by S1-S2 protocol, with the maximum slope of 2.2 for the APD restitution curve and 1.1 m/s^2 for the CV restitution curve.

A representative 1D simulation results with PCL = 320ms was shown in Fig S3.2. The evoked action potential propagation along the strand was colour mapped and plotted in the space-time domain, in which distinctive stranding waves was observed (Fig S3.2A). Although the standing waves were more pronounced in CRN model, the mechanism of generating this functional spatial heterogeneity is the same as demonstrated in the rabbit ventricular cell model as shown in the main manuscript. As shown by the time courses of the computed APD and CV near the stimulus region (a red line marked by **a** in Fig S3.2A) in Fig S3.2B (i-ii), large APD is correlated to a fast CV, whilst a small APD is correlated to a slow CV. Such slow conduction corresponding to small APD led to a conduction delay, allowing other part of the tissue more time to recover from a previous excitation. When the small AP excitation wave reached the more recovered part of tissue, it became large and conducted relatively fast until the excitation wave reaches the refractory tail of the previous excitation again. With time, it developed into a large scale spatial-temporal heterogeneity, which was characterised by the spatial distribution of the APD and CV from beat to beat as illustrated in Fig S3.2C(i-ii). Within the same strand, both concordant and discordant alternans were observed depending on the registration spatial-scale as shown in Fig S3.2D.

2D simulations in homogenous tissue with isotropic and anisotropic conduction was further carried out to see how discordant alternans spontaneously transit into re-entries. Supra-threshold stimuli were given at the left-bottom corner with a PCL of 320ms, which led to AP alternans at cellular level and discordant alternans in 1D simulations. As shown in Fig S3.3, standing waves was observed in isotropic homogenous tissue, but no wave break was observed, which is a similar to the results as shown in Fig 5 in the main manuscript.

In simulations with anisotropic AP conduction shown in Fig S3.4, wave front became unsymmetrical with non-uniform curvature. With the development of functional heterogeneity, the wave front finally caught up the tail of the previous excitation, leading to a spontaneously generated wave break at 1455ms (Fig S3.4A), which further evolved into re-entries till the end of the simulation. It is worth noting that the location of the first wave break is quite near the stimulation site, which means discordant alternans may generate arrhythmia in a small tissue size.

S4. The influence of different prolongations of the recovery time of I_{Na}.

To investigate a possible role of an increased recovery time of I_{Na}, 1D simulations were conducted with varied increase of the inactivation time constant (denoted as h_i) from 0ms to 40ms in reduced (Fig S4 B), normal (Fig S4 C), and increased (Fig S4 D) I_{Na} conditions. For each condition, we recorded the distance between the stimulation site and the first APD node, i.e., the point where distinctive discordant alternans firstly appeared (see the black lines marked in Fig S4 A(i-ii)). If no discordant alternans was observed, the distance would be marked as zero. In the case where discordant alternans was observed, the smaller the distance of the APD node, the more arrhythmogenic the tissue as it required shorter distance for accommodating alternans.

Increase of h_i is correlated in general with a decreased distance of the APD node. As shown in Fig S4 B, at PCL = 135ms and 140ms, the APD node was found to gradually move to the stimulation site with the increase of h_i. However, at PCL = 145ms, the APD node firstly moved close to the stimulation site (compared h_i = 0ms and 10ms), but jumped to a farther location away from the stimulation site with a further h_i increase (compared h_i = 10ms and 20ms). Then, the APD node moved closer to the stimulation site again with the increase of h_i (compared h_i = 20ms, 30ms and 40ms). The same situations were observed at other PCL and S_{gNa}, which were marked by red arrow in Fig S4 B and C. To explain this observation, two representative conditions (with reduced I_{Na} (S_{gNa} = 0.6) at pacing rate of 145ms, h_i=10ms and 20ms respectively) were plotted as space-time plot in Fig S4 A(i-ii), in which a missing beat (the second beat shown in Fig S4 A(ii)) was found in the condition when the APD node jumped to a farther location. Since the generation of discordant alternans is correlated with cardiac memory, the missing beat disturbs the previous regularity and resulted in the APD node relocated to a new point. Except for the APD node relocation marked by the red arrow, all results showed a shortened APD node distance with longer I_{Na} recovery time, suggesting a facilitative role of impaired I_{Na} inactivation in generating discordant alternans.

S5. Role of normal I_{Na} in arrhythmogenesis

Further simulations were carried out to study possible roles of inhomogeneity and anisotropy in the tissue with normal I_{Na} in the spontaneous transition from discordant alternans to arrhythmias. Results shown in Fig S5.1 are obtained from an inhomogeneous 2D tissue model (inhomogeneity was simulated by assigning 10% of cell population as dead cells illustrated by white nodes in Fig S5.1A) with S_{gNa} = 1. In the model, wave-break occurred at time when the wavefront almost caught up with the tail of the previous excitation (Fig S5.1A, screenshot of 1875ms), leading to the formation of re-entries, which sustained through the duration of the simulation (4s, Fig S5.1B). This was in contrast to the result in the homogeneous tissue model with S_{gNa} = 1, in which though obvious standing waves were observed in 1D simulations (Fig 4B in the main MS), and functional heterogeneity manifested as small excitation-refractory islands were generated in 2D model (Fig 5B(i) in the main MS), but no wave break was seen. There is also different to the case in a homogeneous tissue model with S_{gNa}=1.5, where spontaneous transition from alternans to entry was observed (Fig 6 in the main MS). Therefore,

in tissue model without impaired I_{Na}, tissue inhomogeneity plays an important role in promoting the transition from alternans to arrhythmias; but in the tissue with impaired I_{Na} such tissue inhomogeneity is not a necessary condition for facilitating a spontaneous transition from alternans to reentry.

In the 2D anisotropic but homogeneous tissue model with $S_{gNa} = 1$ (with a 2:1 ratio of CV along and perpendicular to the fibre, which was shown by black lines in the top-left panel of Fig S5.2A), no wave break was generated (Fig S5.2A). This is different to the result as shown in Fig 7 in the main MS, where impaired I_{Na} promoted a spontaneous transition from alternans to reentry in homogeneous, but anisotropic tissue. Once again, this result suggests that an important role of impaired I_{Na}, and tissue anisotropy alone is not sufficient to generate the spontaneous transition from alternans to reentry in the tissue with normal amplitude of I_{Na}.
