# Supplementary material for: Mechanistic insight into spontaneous transition from cellular alternans to arrhythmia—A simulation study
Source: PLoS Comput Biol. 2018 Nov 30;14(11):e1006594. doi: 10.1371/journal.pcbi.1006594 (PMC6291170; doi:10.1371/journal.pcbi.1006594)
Supplement: S1 Fig — (PDF) [file pcbi.1006594.s002.pdf]

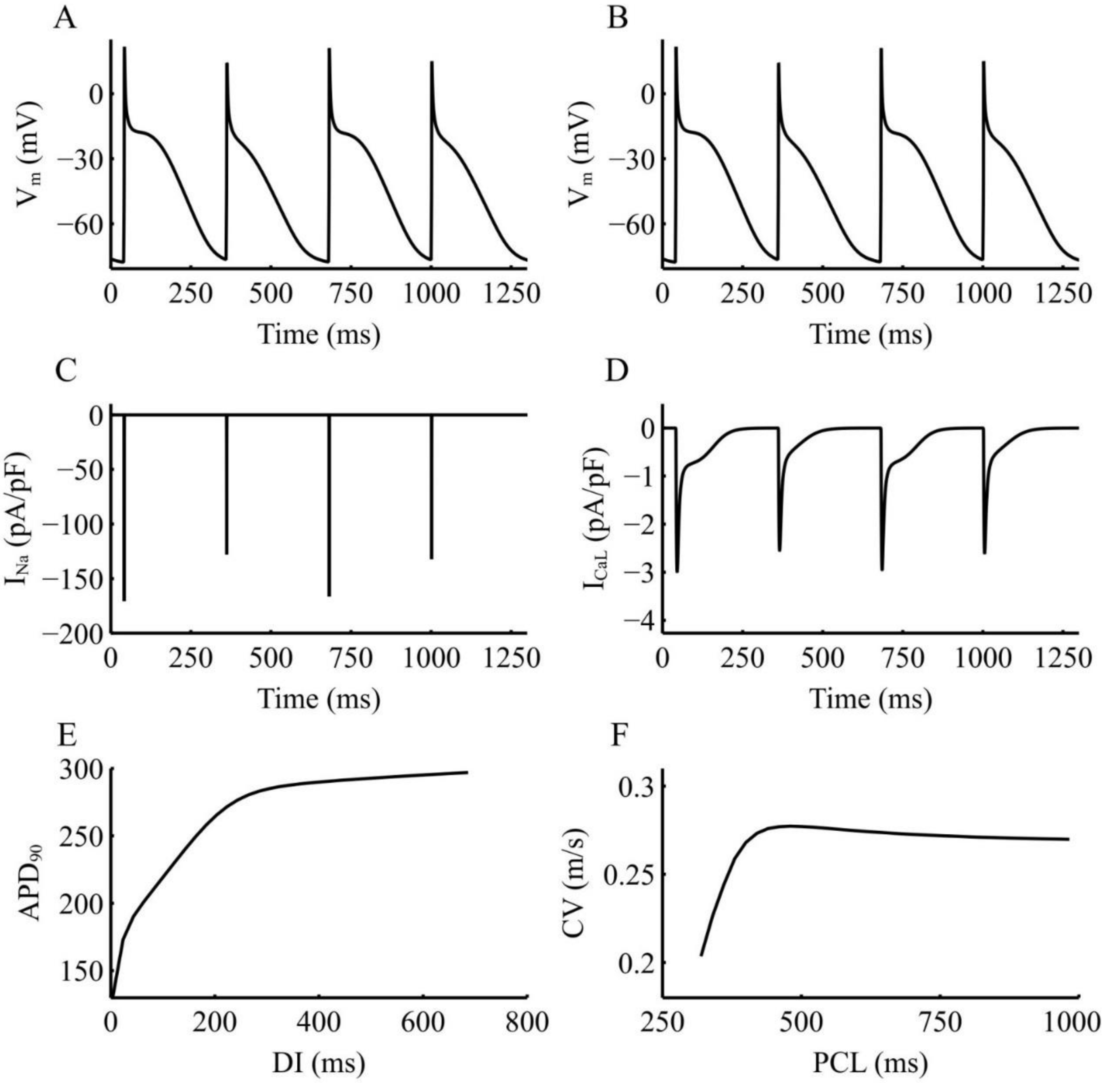

Fig S3.1 Cellular model properties of CRN model. (A-B) Representative AP alternans generated CRN model at PCL = 320ms. (C) Recorded I_{Na} current traces during AP alternans. (D) Recorded I_{CaL} current traces during AP alternans. (E) APD restitution curves using S1-S2 protocol of the single cell models. (F) CV restitution curve computed by using the S1-S2 protocol.
