# Supplementary material for: Mechanistic insight into spontaneous transition from cellular alternans to arrhythmia—A simulation study
Source: PLoS Comput Biol. 2018 Nov 30;14(11):e1006594. doi: 10.1371/journal.pcbi.1006594 (PMC6291170; doi:10.1371/journal.pcbi.1006594)
Supplement: S2 Fig — (PDF) [file pcbi.1006594.s003.pdf]

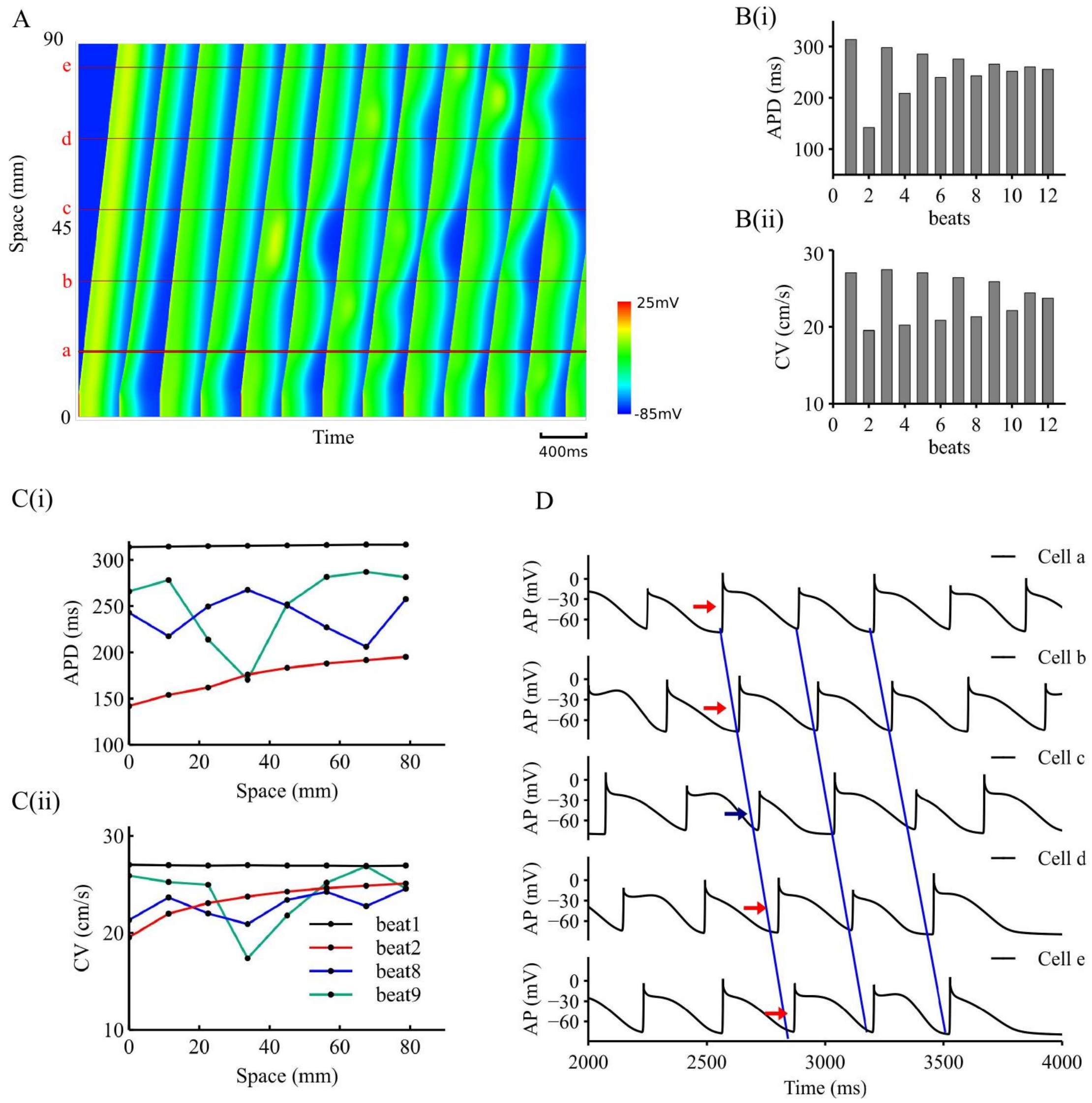

Fig S3.2 Representative 1D simulation results at PCL = 320ms. **(**A) Space-time plot of AP propagation on 1D strand. (B) APD (B(i)) and CV(B(ii)) variations at location **a** (marked in A) dependent on simulated beats. (C) Spatial distributions for APD(C(i)) and CV(C(ii)) for beat 1,2,8,9. (D) Time course traces for location **a**, **b**, **c**, **d**, **e** marked in A. Both concordant and discordant alternans were observed in the strand depending on the spatial scale of observation.
