# Supplementary material for: Mechanistic insight into spontaneous transition from cellular alternans to arrhythmia—A simulation study
Source: PLoS Comput Biol. 2018 Nov 30;14(11):e1006594. doi: 10.1371/journal.pcbi.1006594 (PMC6291170; doi:10.1371/journal.pcbi.1006594)
Supplement: S3 Fig — (PDF) [file pcbi.1006594.s004.pdf]

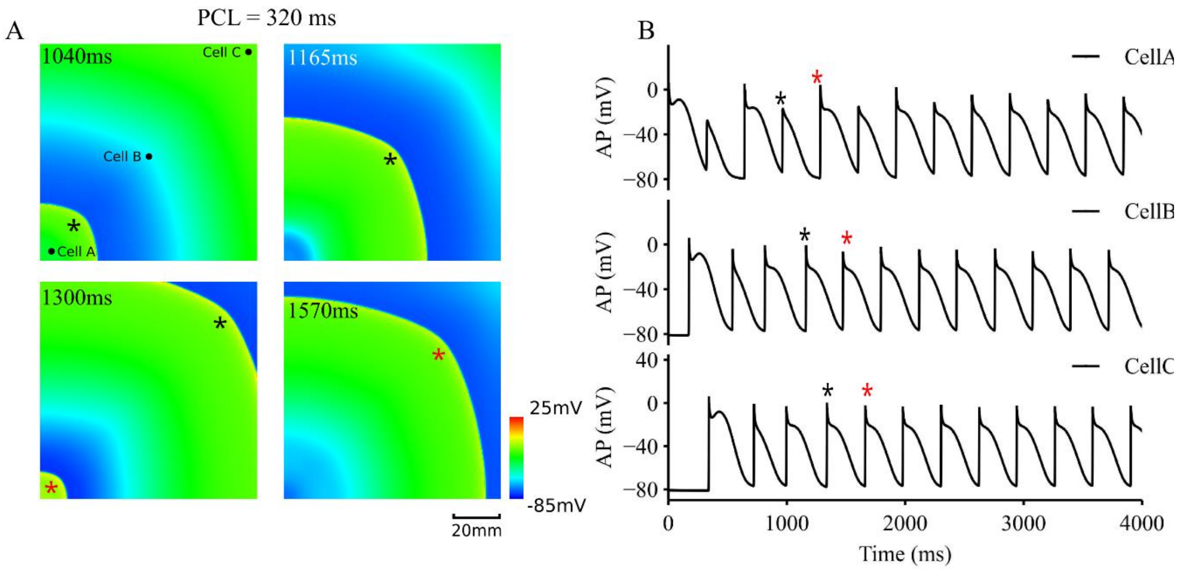

Fig S3.3 Snapshots of conduction of AP alternans in 2D homogenous tissue and time series of APs. (A) Snapshots of excitation waves in an anisotropy tissue with normal I_{Na}. PCL = 320ms. (B) Time series of APs recorded from 3 different registration sites, cell A, B and C as marked by black dots in the top-left panel of A. Black and red stars marked two consecutive stimulus pulses.
