# Supplementary material for: Mechanistic insight into spontaneous transition from cellular alternans to arrhythmia—A simulation study
Source: PLoS Comput Biol. 2018 Nov 30;14(11):e1006594. doi: 10.1371/journal.pcbi.1006594 (PMC6291170; doi:10.1371/journal.pcbi.1006594)
Supplement: S5 Fig — (PDF) [file pcbi.1006594.s006.pdf]

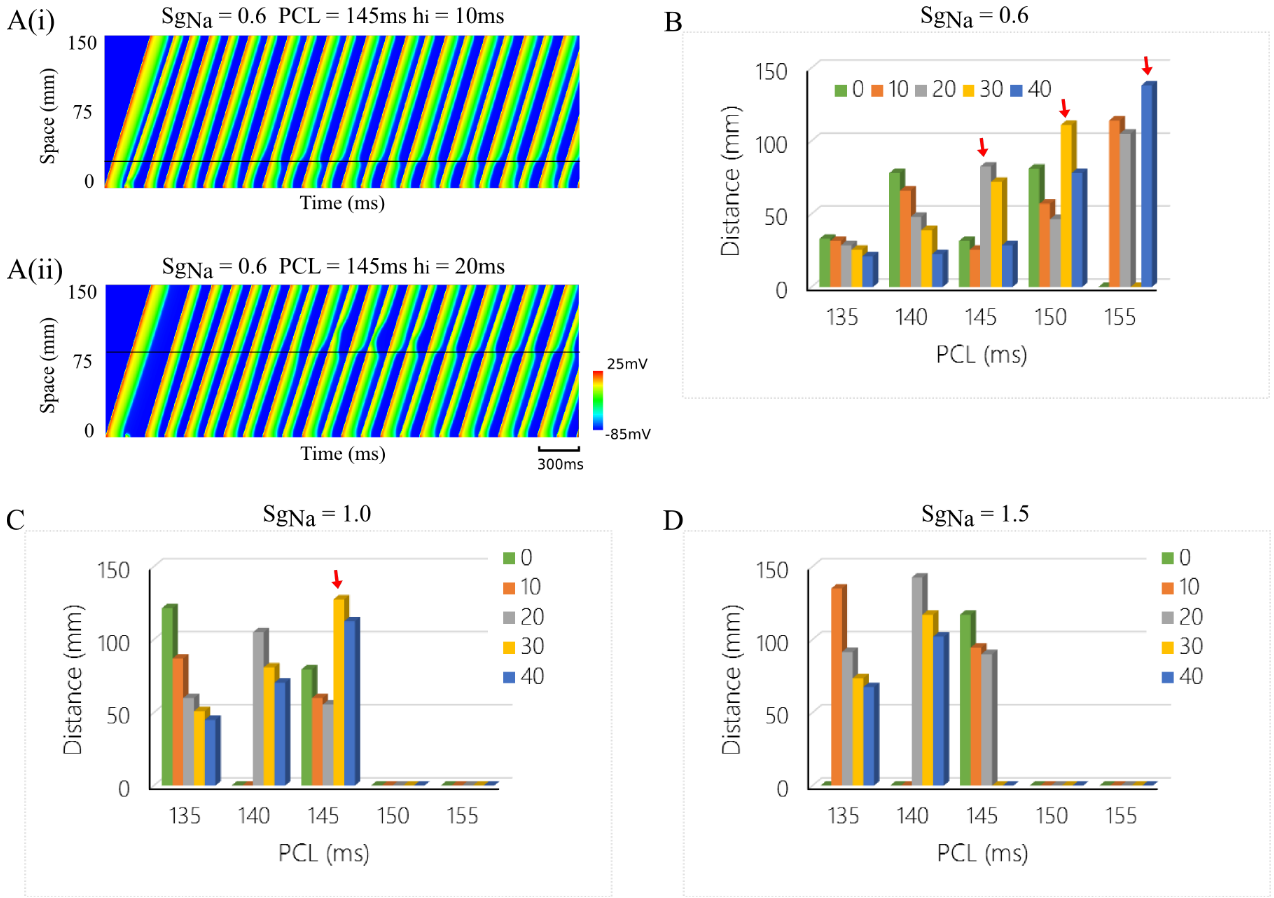


Fig S4 Role of $I_{Na}$ recovery time in the location of APD node. (A) Representative 1D space-time results with $S_{gNa}$=0.6, PCL = 145ms, $h_i$=10ms (i) and 20ms (ii). (B) Statistic APD node distances from stimulation site at different recovery time prolongations and PCLs with reduced $I_{Na}$. (C) Statistic APD node distances from stimulation site at different recovery time prolongations and PCLs with normal $I_{Na}$. (D) Statistic APD node distances from stimulation site at different recovery time prolongations and PCLs with increased $I_{Na}$. Red arrow marked the APD node relocated conditions.
