# Supplementary material for: Mechanistic insight into spontaneous transition from cellular alternans to arrhythmia—A simulation study
Source: PLoS Comput Biol. 2018 Nov 30;14(11):e1006594. doi: 10.1371/journal.pcbi.1006594 (PMC6291170; doi:10.1371/journal.pcbi.1006594)
Supplement: S6 Fig — (PDF) [file pcbi.1006594.s007.pdf]

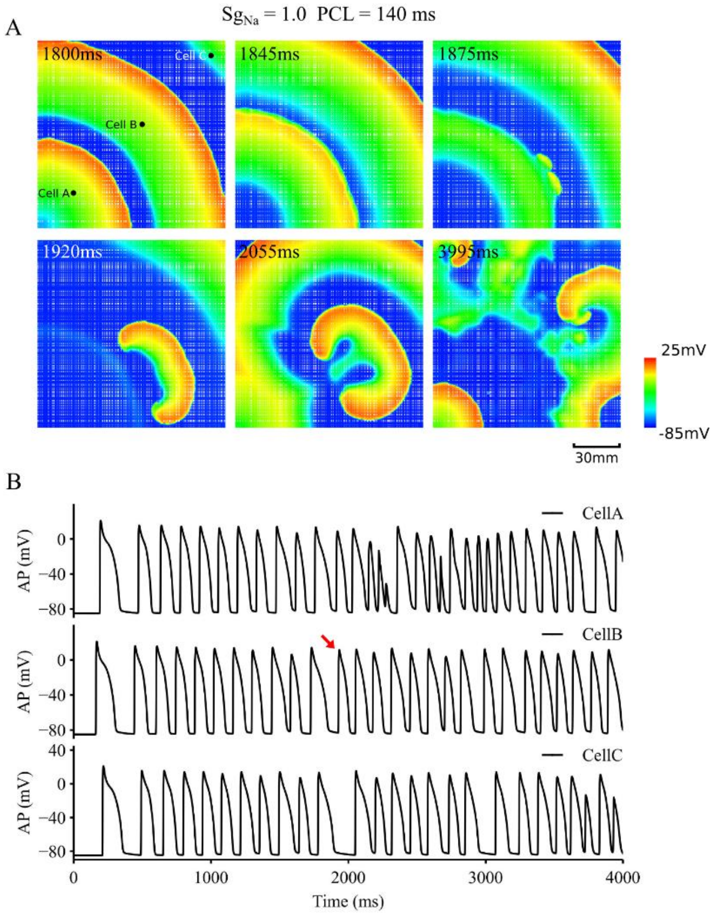

Fig S5.1 Conduction of AP alternans in 2D inhomogeneous tissue with normal I_{Na} (S_{gNa}=1) and time series of APs. (A) Snapshots of excitation waves in an inhomogeneous tissue with PCL = 140ms. (B) Time series of APs recorded from 3 different registration sites, cell A, B and C as marked by black dots in the top-left panel of (A). Red arrow: marking for beginning of re-entry. Inhomogeneous tissue was simulated by assigning 10% cells to dead cells randomly in the tissue as shown by white nodes.
